# Molecular docking of C-Jun-N-Terminal Kinase (Jnk) with amino-pyrimidine derivatives

**DOI:** 10.6026/97320630016462

**Published:** 2020-06-30

**Authors:** Krishnamoorthy Meenakumari, Giridharan Bupesh

**Affiliations:** 1 Research and Development Wing, Central Research Laboratory, Sree Balaji Medical College and Hospital (SBMCH), BIHER, Chrompet, Chennai - 600044, India

**Keywords:** JNK3, inhibitors, amino-pyrimidine

## Abstract

It is of interest to document the molecular docking of C-Jun-N-Terminal Kinase (Jnk) (known structure with PDB ID: 1PMN) with amino-pyrimidine derivatives in the context of Alzheimer's Disease (AD). We report the optimal binding features (binding energy, interacting residues, inter atomic hydrogen bonding patterns) of 11 amino-pyrimidine derivatives with Jnk for further consideration.

## Background

Alzheimer's disorder (AD) with defined symptoms is the 6th leading reason of death in the United States [1-2]. AD development range from mild to severe in middle-aged humans to older people detected with cognitive exams [3]. The JNK family of proteins are well studied and documented in the literature [4-14]. JNK3 is known target for AD [15- 17]. It is of interest to document the molecular docking of JNK3 (known structure with PDB ID: 1PMN) with amino-pyrimidine derivatives for further consideration.

## Materials and Methods:

### JNK3 protein:

**Figure 1:**
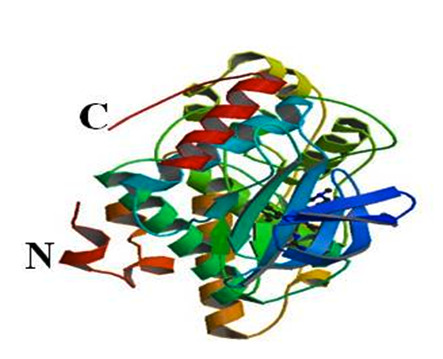
Structure of Jnk3 (PDB ID: 1PMN) drawn using Schrodinger glide

The crystal structure of human (JNK3) (PDB ID: 1PMN) from http://www.rcsb.org/pdb with resolution 2.20 Å is used in this study. The Schrodinger suite was used for energy minimization and optimization of the structure (Figure 1).

### Ligand data:

A series of 11 amino-pyrimidine derivatives from known literature is used in this study ( 1) are drawn using the chemsketch software.

### Molecular docking:

The Maestro Suite was used for molecular docking using known standard procedure.

### Prediction of drug-likeliness data

The Lipinski Rule of 5 validation and ADME (absorption, distribution, metabolism and excretion) data were computed using standard tools.

### ADME toxicity data:

ADME/T data was collected using the QikProp module in Schrodinger.

## Results and discussion:

**Figure 2:**
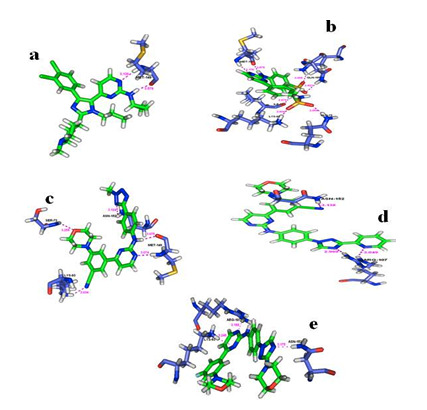
Molecular docking interaction of JNK3 with the amino -pyrimidine derivatives (a) native ligand, (b) ligand 9D, (c) ligand 9G, (d) ligand 9J, (e) ligand 9L

The structure of the target protein JNK3 (PDB ID: 1PMN) at a resolution of 2.20 Å is shown (Figure 1).The names of amino-pyrimidine derivatives used in this study are given in Table 1. The structures of ligands are drawn using the CHEMSKETCH software. Energy minimization was done using the OPLS_AA force field. Data from the High Throughput Virtual Screening (HTVS) using GLIDE HTVS five module with relevant information is given in Table 2. The Four compounds and the native ligand from HTVS have been subjected to Induced Fit Docking (IFD). Figure 2 and Table 3 shows data for the possible conformations of the best ligands with their docking score and GLIDE power. The ADME/T properties of the best compound have been further analyzed by using the QIKPROP tool of the Schrodinger Software. Pharmacodynamics and pharmacokinetics properties of lead compounds were evaluated using the Qikprop tool in Maestro. Ligand 9D, Ligand 9G, Ligand 9J, Ligand 9L show good Glide score. These compounds have high QPlogHERGK+ channels, QPlogPo/w, QPlogKP, QPlogBB and QPlogKhsa values that satisfy the Lipinski's Rule of Five (Table 4). Data show that Ligand 9D have better permeation rate (Table 4). The ligand 9D has optimal interactions with the catalytic residues (LYS 93, GLN 75, GLN 155, MET 149, GLN 155, MET 149, ILE 70) with high binding ability for further in vitro and in vivo studies. This data is highly relevant to in the activation of JNK [19,20].

## Conclusions:

Docking studies showed that ligand 9D (4-(4-(4-(Methyl sulfonamido) phenyl) pyrimidin 2yl-amino) benzene-sulfonamide) have the best docking score (-12.193) and Glide energy (-68.711) compared to the native ligand. It also has strong hydrogen bonding interaction at the peripheral site residue MET 149 and showed a similar binding mode of interaction with MET 149 in the native ligand. Hence, ligand 9D has optimal interactions with the catalytic residues (LYS 93, GLN 75, GLN 155, MET 149, GLN 155, MET 149, ILE 70) with high binding ability for further in vitro and in vivo studies.

## Figures and Tables

**Table 1:** Chemical names for Aminopyrimidine derivatives

ENTRIES	CHEMICAL NAME
9(A)	4-(4-Phenylpyrimidin-2-ylamino)bemzamide
9(B)	N-(4-(1H-1,2,4-Triazol-1-yl)phenyl)-4-phenylpyrimidin-2-amine
9(C)	N-(4-(1H-1,2,4-Triazol-1-yl)phenyl)-4-phenylpyrimidin-2-amine
9(D)	4-(4-(4-(Methylsulfonamido)phenyl)pyrimidin 2ylamino) benzene-sulfonamide
9(E)	N-(4(-2-(4-(1H-1,2,3-Triazol-1-yl)phenylamino)primidin-4-yl)-phenyl)methanesulfonamide
9(F)	N-(4-(3-Methyl-1H-1,2,4-Triazol-1-yl)phenyl)-4-(3-morpholino-phenyl)pyrimidin-2-amine
9(G)	3-(2-(4-(3-Methyl-1H-1,2,4-Triazol-1-yl)phenylamino)pyrimidin-4-yl)-5-morpholinobenzonitrile
9(I)	4-(3-Morpholinophenyl)-N-(4-(3(pyridine-3-yl)-1H-1,2,4-triazol-1-yl)phenyl)pyrimidin-2-amine
9(J)	3-Morpholino-5-(2-(4-(3-(pyridine-2-yl)-1h-1,2,4-triazol-1-yl)-phenylamino)pyrimidin-4-yl)benzonitrile
9(K)	4-(3-Fluoro-5-morpholinophenyl)-N-(4-(3-(4-methylpiperazin-1-yl)-1H1,2,4-triazol-1-2-amine
9(L)	4-(3-Fluoro-5-morpholinophenyl)-N-(4-(3-morpholino-1H-1,2,4-triazol-1-yl)phenyl)pyrimidin-2-amine
Native Ligand	Cyclopropyl-{4-[5-(3,4-Dichlorophenyl)-2-[(1-Methyl)-Piperidin]-4-yl-3-Propyl-3H-Imidazol-4-yl]-Pyrimidin-2-yl}Amine

**Table 2:** High Throughput Virtual Screening Results of 11 Ligands (Aminopyrimidine derivatives) against the Target c-Jun terminal kinases (JNK3).

Ligands	Docking Score	Glide Energy Kcal/mol	Hydrogen Bond D-H...A	Distance Å
Ligand 9J	-8.62	-85.68	O-H...O (THR 199)	3.29
			(GLN 92) N-H...O	2.89
Native ligand	-7.28	-68.27	(HIS 64) N-H...O	2.68
			(GLN 92) N-H...O	2.96
			(MET 109) N-H...N	3.36
			N-H...O (THR 199)	2.07
Ligand 9L	-7.03	-53.28	N-H...O (GLU 71)	3.12
Ligand 9K	-6.08	-36.27	N-H...O (PRO 201)	3.52
			N-H...O(ASP 112)	3.07
Ligand 9E	-5.2	-27.2	-	-
Ligand 9C	-5.62	-22.76	-	-
Ligand 9D	-6.38	-43.18	(LYS 53) N-H...N	3.81
			(HIS 94) N-H...O	2.55
Ligand 9G	-5.08	-38.97	N-H...O (PRO 201)	3.25
			N-H...O (THR 200)	3.61
Ligand 9A	-5.89	-21.06	(THR 200) N-H...O	2.29
			(THR 200) O-H…O	3.55
Ligand 9B	-5.28	-20.84	-	-
Ligand 9I	-6.02	-19.27	(THR 199) N-H...O	3.05
Ligand 9F	-4.97	-23.31	(THR 200) O-H...O	3.78

**Table 3:** Induced Fit Docking Results of the 4 ligands and the Native Ligand against the Target c-jun-N-terminal kinase (JNK)

Poses	D-H...A	Distance Å	Docking Score Kcal/mol	Glide Energy Kcal/mol
Native Ligand	(GLN 75) N-H...N	2.852	-11.103	-67.444
	(MET 149) N-H...N	3.126		
	N-H...O (MET 149)	3.576		
Ligand 9D	(LYS 93) N-H...O	2.604	-12.193	-68.711
	(GLN 75) N-H...O	2.954		
	(GLN 155) N-H...O	2.921		
	N-H…O (MET 149)	3.575		
	(GLN 155) N-H...O	3.005		
	(MET 149) N-H...N	3.176		
	N-H…O (ILE 70)	2.876		
Ligand 9G	(LYS 93) N-H...N	3.034	-10.043	-56.159
	(MET 149) N-H...N	3.074		
	N-H...O (MET 149)	3.075		
	(SER 72) N-H...O	3.259		
	(ASN 152) N-H...O	3.124		
Ligand 9J	(ARG 107) N-H...H	3.049	-8.717	-68.353
	(ARG 107) N-H...H	2.999		
	(ASN 152) N-H...N	3.138		
Ligand 9L	(ARG 107) N-H...N	3.188	-7.827	-56.054
	(ASN 152) N-H...N	3.175		
	(LYS 93) N-H...N	3.248		

**Table 4:** ADMET prediction of Aminopyrimidine derivatives of the selected compound with best Glide Score

Ligands	QP log Po/w	QP log HERG	QPP Caco (nm/s)	QP log BB	QPP MDCK (nm/s)	Q Plog Kp
Ligand 9D	5.344	-6.278	56.395	-3.658	50.927	-3.524
Ligand 9G	2.605	-2.257	20.512	-2.348	28.452	-4.201
Ligand 9J	2.328	-3.638	19.024	-1.8	10.021	-2.532
Ligand 9L	2.687	-2.263	24.598	-1.796	18.967	-4.777
